# Chemical Synthesis of Proanthocyanidins *in Vitro* and Their Reactions in Aging Wines

**DOI:** 10.3390/molecules13123007

**Published:** 2008-12-04

**Authors:** Fei He, Qiu-Hong Pan, Ying Shi, Chang-Qing Duan

**Affiliations:** Center for Viticulture and Enology, College of Food Science & Nutritional Engineering, China Agricultural University, Beijing, 100083, P.R. China; E-mails: wheyfey@yahoo.com.cn (F. H.); panqiuhong2007@vip.sohu.com (Q-H. P.); shiy@cau.edu.cn (Y. S.)

**Keywords:** Proanthocyanidins, Condensed Tannins, Chemical Synthesis, Stereoselective Synthesis, Flavanols, Anthocyanins, Wine.

## Abstract

Proanthocyanidins are present in many fruits and plant products like grapes and wine, and contribute to their taste and health benefits. In the past decades of years, substantial progresses has been achieved in the identification of composition and structure of proanthocyanidins, but the debate concerning the existence of an enzymatic or nonenzymatic mechanism for proanthocyanidin condensation still goes on. Substantial attention has been paid to elucidating the potential mechanism of formation by means of biomimetic and chemical synthesis *in vitro*. The present paper aims at summarizing the research status on chemical synthesis of proanthocyanidins, including non-enzymatic synthesis of proanthocyanidin precursors, chemical synthesis of proanthocyanidins with direct condensation of flavanols and stereoselective synthesis of proanthocyanidins. Proanthocyanidin-involved reactions in aging wines are also reviewed such as direct and indirect reactions among proanthocyanidins, flavanols and anthocyanins. Topics for future research in this field are also put forward in this paper.

## 1. Introduction

Proanthocyanidins (PAs), also known as condensed tannins, are a group of important secondary metabolites synthesized via the flavonoid pathway. They are widespread throughout the plant kingdom where they display multiple biochemical properties, mainly involving interactions with proteins, the chelation of metals and antioxidant activity, which are the basis of their various protective functions for plants [1,2,3,4,5]. Meanwhile, PAs are also widely present in plant foodstuffs or made from plants, especially in wines, where they provide the bitter flavor and astringency and have a significant influence on the color alteration [6,7]. Recently, much attention has been drawn to the so-called “French Paradox”, which may be partially attributed to the anti-atherogenic activity of PAs in red wine [8]. From then on, more and more efforts have been dedicated to research seeking their potential beneficial effects on human health [9].

In recent years, a series of key enzymes, transcription factors and intracellular transport factors involved in the biosynthesis of PAs have been identified by genetic and molecular genetic approaches [10]. However, the final “enzymes” which catalyze the polymerization reaction of flavanols to form PAs and the exact mechanism of how these flavanol units assemble *in vivo* remains unclear. The debate whether the polymerization of PAs takes place through a complex enzymatic pathway or through direct non-enzymatic condensation has been ongoing for many decades and continues, even nowadays [11]. Therefore, a unilateral genetic or molecular genetic research seems to be inadequate and chemical or biomimetic synthesis of PAs with direct condensation of flavanols *in vitro* should not be neglected. 

Further more, although flavanols and their oligomers or polymers are widespread in plants, their isolation usually requires time-consuming procedures and both quality and reproducibility are usually unsatisfactory. Meanwhile, potential substrates of PA condensation are not all commercially available due to their potential multiform stereochemistry and the instability conferred by their strong reducibility, which cause trouble to the studies on the mechanism of PA polymerization and the improvement of their biological activities [12]. To overcome these difficulties, great efforts have been devoted into the chemical synthesis of flavanols and PAs. On the other hand, as one main part of wine polyphenolic compounds, PAs are transferred from the solid parts of the grape (skins, seeds, and stems) into the must during winemaking operations (crushing, maceration, and fermentation) [13]. And during the wine aging, PAs, flavanols and anthocyanins are rather unstable compounds that undergo various types of chemical reactions, resulting in changes of color and taste [14,15]. Besides, PAs also participate in oxidative browning reactions, in haze formation and interactions with proteins, and in direct or indirect condensation reactions in wine that give the fundamental support to the potential polymerization mechanism for PAs and the valuable inspiration to researches in this field [16,17,18].

In the present paper, we summarize the research status on biomimetic and chemical synthesis of PAs, including non-enzymatic synthesis of PA precursors, biomimetic or chemical synthesis of PAs with direct condensation of flavanols and stereoselective synthesis of PAs. PA-involved reactions in aging wines are also reviewed such as direct and indirect reactions among PAs, flavanols and anthocyanins, which can help us find out the potential mechanism of PA synthesis. The anticipated future trends in this field are also discussed.

## 2. Chemical synthesis of proanthocyanidins

### 2.1 Nonenzymatic synthesis of proanthocyanidin precursors

Generally, PAs are synthesized as oligomers and polymers made up of elementary flavan-3-ol units. However, both (2*R*,3*S*)-flavan-3-ols [mainly (+)-catechins] and (2*R*,3*R*)-flavan-3-ols [mainly (-)-epicatechins], as well as (2*R*,3*S*,4*S*)-flavan-3,4-diols are considered as the potential direct precursors of PAs [19].

**Scheme 1:**
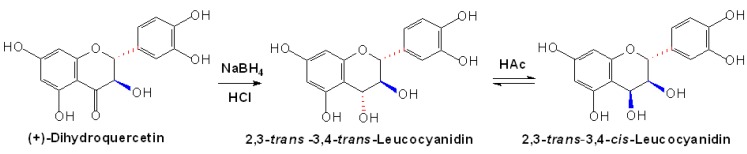
Non-enzymatic reduction of (+)-dihydroquercetin to (±)-leucocyanidin [21].

In the biosynthesis of flavan-3,4-diols (leucoanthocyanidins: leucopelargonidin, leucocyanidin and leucodelphinidin, respectively), dihydroflavonol 4-reductase (DFR) can stereospecificly convert (+)-dihydroflavonols to their corresponding (2*R*,3*S*,4*S*)-flavan-3,4-diols via a NADPH dependent mechanism [20]. This enzymatic reaction was firstly demonstrated in crude soluble protein extracts from cell or tissue cultures of Douglas fir (*Pseudotsuga menziesii*) [21]. Meanwhile, the non-enzymatic reduction of (+)-dihydroquercetin with NaBH_4_ to produce two presumably isomeric flavan-3,4-diols, as shown in Figure 4, was also detected, with one being methanol-soluble and similar to the enzymatic product, presumed as 3,4-*cis* configuration (2*R*,3*S*,4*S*), and another being water-soluble and different from nature product, presumed as 3,4-*trans* configuration (2*R*,3*S*,4*R*). However, both of the two kinds of flavan-3,4-diols were highly unstable in acid media, and they can both react with (+)-catechin to form PA oligomers [21]. Although yields of this non-enzymatic method are poor, it still offers us the reliable production of flavan-3,4-diols for further study nowadays. Recently, based on the previous reduction method, an efficient conversion of (+)-catechin to 2,3-*trans*-3,4-*trans*-leucocyanidin was developed through several steps, which involved benzylation, acetoxylation, hydrolysis, oxidation, deprotection and stereoselective reduction, showing us an alternative substrate to obtain flavan-3,4-diols [22].

**Scheme 2:**
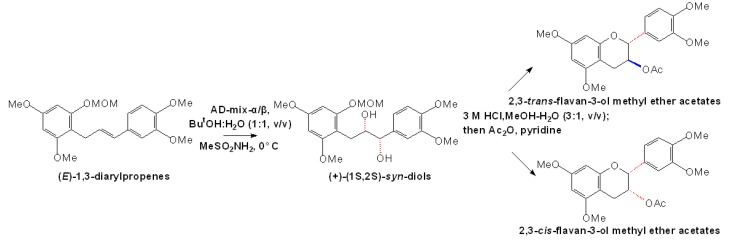
Synthesis strategy for flavan-3-ol derivatives with non-flavonoid substrates [26].

Similar conversion of (+)-dihydroquercetin to flavan-3-ols or flavan-3,4-diols to flavan-3-ols was also demonstrated by using crude extracts of various plants, such as barley testa/pericarp, Douglas fir and sainfoin (*Onobrychis viciifolia*) leaves, which provide direct evidences to elucidate the function of DFR and leucoanthocyanidin reductase (LAR) [23,24,25]. In addition to the enzymatic conversions, chemical synthetic flavan-3-ols by using non-flavonoid as substrates were also identified. A series of polyoxygenated (*E*)-1,3-diarylpropenes obtained from (*E*)-*retro*-2-methoxymethylchalcone methyl ethers were converted, through asymmetric dihydroxylation and subsequent acid-catalyzed cyclization, to produce *trans*- and *cis*-flavan-3-ol methyl ether acetates, essentially enantiopure and in productive, which are available in quantities sufficient for preparative purposes, as shown in Scheme 2 [26].

### 2.2 Chemical synthesis of proanthocyanidins with direct condensation of flavanols or their derivatives

Condensation of either (+)-catechin or (-)-epicatechin with leucocyanidin derived from (+)-dihydroquercetin was first conducted by Creasey and Swain in 1960s, to synthesize procyanidins (PAs only composed (+)-catechins and/or (-)-epicatechins), which offered a fundamental support to the hypothesis of quinone methides or carbocations for PA synthesis [27]. However, due to the limitation of the experimental methods and equipment at that time, this research could not figure out the unambiguous configuration of the products, as well as the exact mechanism of PA polymerization. Since then, a lot of efforts have been put into chemical or biomimetic synthesis of procyanidins and a number of papers dealing with this kind of investigations have been published, which greatly facilitate our understanding of the potential mechanism for PA condensation.

In the 1980s, researchers found that the reaction between epicatechin-(4*β*)-phenyl sulphide and leucocyanidin under alkaline pH was more rapid than under acidic pH, suggesting that the biosynthesis of condensed tannins might occur through a quinone methide rather than a carbocation intermediate and giving us a new commentation about the hypothesis of quinone methides or carbocations [28]. Furthermore, the first branched procyanidin trimer, epicatechin-(4β→8)-catechin-(6→4β)-epicatechin was yielded in high ratio than its linear analogues, suggesting that procyanidin polymers may be highly branched in natural products [29]. Then, it was proposed that a transformation from (2*R*,3*R*)-2,3-*trans*-dihydroflavonols to (2*R*,3*S*)-2,3-*cis*-PAs could be achieved by tautomerism between quinone methide and flav-3-en-3-ol intermediates, further supporting the hypothesis of quinone methides or carbocations [30]. 

Concurrently, researchers tried to determine the stereochemistry of PA biomimetic synthesis. By using the electrophilic flavanyl-4-carbocations generated from flavan-3,4-diols and the nucleophilic flavan-3-ols as the substrates, a direct approach to synthesize [4,6]- and [4,8]-biflavanoids (procyanidins) was developed at ambient temperatures and under mildly acidic aqueous conditions. The stereospecificity of the reaction was found to be conditioned not only by the stereochemistry of the flavan-3,4-diols, but also by the nucleophilicity and the regiospecific course of the flavan-3-ols [31]. Additionally, what supported this assumption about the stereochemistry of PA synthesis is the finding that following the enzymatic and nonenzymatic reduction of (+)-dihydroquercetin to flavan-3,4-diols, the flavan-3,4-diols could be further condensed with added (+)-catechin to form the all-*trans* dimers and trimers of (+)-catechin under acid conditions [21].

**Scheme 3:**
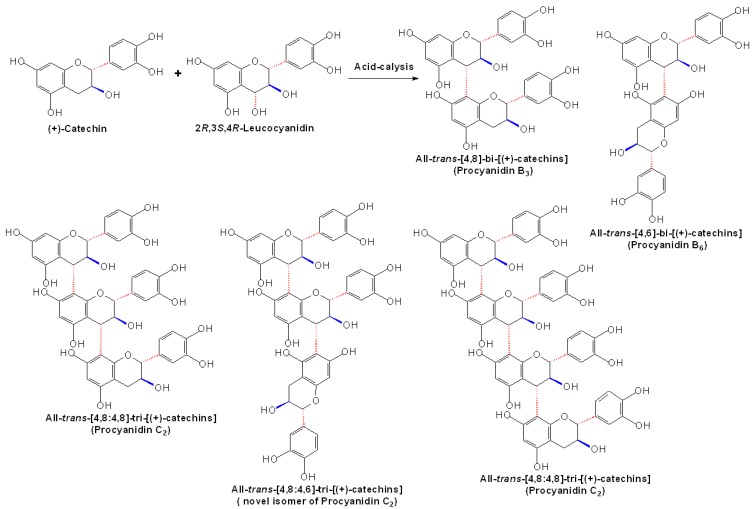
Condensation between molar equivalents of (2*R*,3*S*,4*R*)-leucocyanidin and (+)-catechin [32].

Besides, in the molar equivalent condensation between synthetic (2*R*,3*S*,4*R* or 4*S*)-leucocyanidin and (+)-catechin, the reaction proceeded rapidly at mild acidic pH under ambient conditions to produce a series of all*-trans* oligomeric procyanidins, including two dimers, procyanidins B3, B6, two trimers, procyanidin C2 and its novel isomer, as well as one tetramer, as shown in Scheme 3 [32]. However, in the condensation between (+)-leucocyanidin [(2*R*,3*S*,4*S*)-2,3-*trans*-3,4-*cis*-leucocyanidin] and (-)-epicatechin, besides the products mainly containing [4,8]-2,3-*trans*-3,4-*cis*-procyanidin units, a ‘trimer’ of mixed stereochemistry containing the terminal moieties of 2,3-*trans*-3,4-*trans*-procyanidin configuration was also detected by ^1^H-NMR spectroscopy [33]. Therefore, the stereochemistry of PA biomimetic synthesis seemed to be more complicated than ever thought possible.

**Scheme 4:**
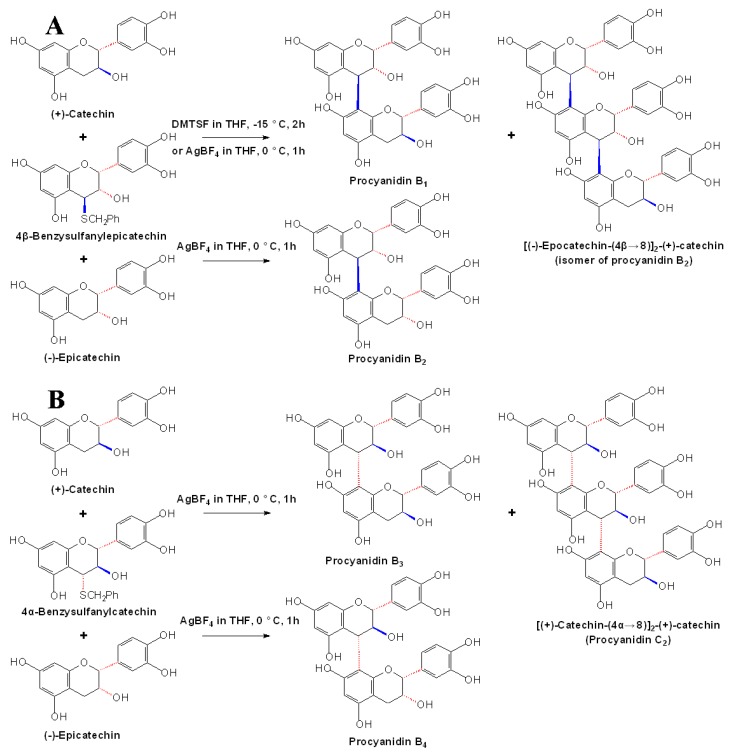
Thiophilic Lewis acids activated chemical synthesis of PA oligomers [34].

However, in these semi-synthetic methods which evolved coupling of electrophilic C4-substituted flavan-3-ols under acidic or basic conditions, the interflavan linkages (IFL) in the products were usually labile under such reaction conditions [28,31]. Recently, the thiophilic Lewis acids dimethyl(methylthio)sulfonium tetrafluoroborate (DMTSF) and silver tetrafluoroborate (AgBF_4_) were used to activate C4-thioethers of flavan-3-ols towards carbon nucleophiles to generate the IFL of procyanidins under neutral conditions. By using these Lewis acids and 4β-benzylsulfanylepicatechin and 4α-benzylsulphanylcatechin, Steynberg *et al*. obtained a succession of stable procyanidin oligomers, including procyanidin B1, B2, B3, B4, C2 and its trimeric analogue, as shown in Scheme 4 [34]. Anyway, it could be recognized as a beginning of the stereoselective synthesis of PAs.

A) condensation between flavan-3-ols and 4β-benzylsuifanylepicatechin; B) condensation between flavan-3-ols and 4α-benzylsulphanylcatechin.

Besides the well-known procyanidins, propelargonidins and prodelphinidins, other PAs that have different hydroxylation patterns were also chemically synthesized, including proguibourtinidins (3,4’,7-hydroxyl), profisetinidins (3,3’,4’,7-hydroxyl), prorobinetinidins (3,3’,4’,5’,7-hydroxyl), proteracacidins (4’,7,8-hydroxyl), promelacacidins (3’,4’,7,8-hydroxyl), proapigeninidins (4’,5,7-hydroxyl) and proluteolinidins (3’,4’,5,7- hydroxyl), etc [35,36,37,38]. As early as in 1970s, Botha *et al*. first synthesized 4,8:4,6-linked all-*trans*- and 2,3-*trans*-3,4-*trans*:2’,3’-*trans*:2’’,3’’-*trans*-3’’,4’’-*cis*-bi[(-)-fisetinidol]-(+)-catechins and recognized their distribution in Nature [39]. From then on, as an interesting research field of heterocyclic chemistry, it has drawn considerable attention and a great deal of achievements have been obtained, which were summarized in reviews by the research group of Ferreira [40,41,42].

### 2.3 Stereoselective synthesis of B-type procyanidins

Although the chemical or biomimetic synthesis strategies with flavanols could offer us a direct way to investigate the polymerization mechanism of PAs, they still have some deficiencies. On one hand, the direct substrates for biomimetic synthesis, flavan-3,4-diols or its derivatives are usually unstable and are readily oxidized in biological extracts. As a result, they have not been identified directly in plant extracts and are not commercially available, restricting their usage in PA synthesis. On the other hand, the products of biomimetic synthesis are usually obtained as a complex mixture of structurally related compounds which are difficult to isolate. Consequently, more and more research was planned to develop efficient synthetic methods leading to PAs, especially the procyanidins, with a high level of purity and stereospecificity. The commonly used synthetic strategies invented towards this purpose mainly include the mechanism of the phenol protection, the Lewis acid (or its substitutes) activation and the bromo-capping of the C8 position.

The benzyl moiety is widely used as a phenol protecting group in the stereoselective synthesis of PAs and other organic chemicals which contain phenol groups. It can be removed in the final step by a hydrogenolysis reaction which is usually easy, clean and efficient. Several other commonly used phenol protecting groups include methoxymethyl, benzyloxymethyl, tosyl and benzenesulfonyl, but not all these could be removed from the final products in a similar scheme [43]. In stereoselective synthesis of procyanidins, (+)-catechins are usually alkylated with benzyl bromide to form mono- and di-C-benzyl derivatives with free hydroxyl groups under different conditions, which can be further used as the potential substrates for procyanidins synthesis [44]. Generally, 5,7,3’,4’-tetrabenzylcatechin or 5,7,3’,4’-tetrabenzylepicatechin are the most widely used benzylated flavan-3-ols.

Another main procedure for procyanidins synthesis is Lewis acid activation, which includes trimethylsilyl trifluoromethanesulfonate (TMSOTf), AgBF_4_ and TiCl_4_ for (-)-epicatechin and (+)-catechin dimmer, SnCl_4_ and BF_3_·Et_2_O for (+)-catechin oligomer, etc [45,46,47]. Usually, as a result of Lewis acid activation, a hydroxyl group at the C4 position is first formed in the derivatives of flavan-3-ols, which could be used as the electrophiles. Nowadays, the stereospecificity of Lewis acid-activated procyanidins polymerization has been further improved by using different chemicals or their substitutes. 

Bromo-capping of the C8 position is another specified method for phenol protection, which has been widely used to avoid the formation of self-condensation products and multiple undesired reactions [48]. As early as the 1980s, through selective bromination and debromination reactions and via lithio-analogues, Hundt and Roux obtained pairs of C8 and C6 functionalized (Br, OH, OAc, CO_2_Me, and CH_2_Me groups) 3′ ,4′ ,5,7-tetra-*O*-methyl-(+)-catechins, which could be used for stereoselective synthesis of PAs [49]. 

**Scheme 5:**
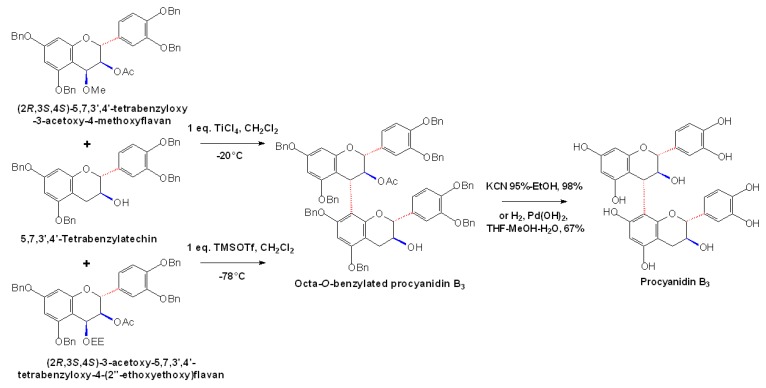
Stereoselective synthesis of procyanidin B3 [51].

In 2002, Saito *et al*. developed a method of stereoselective synthesis of Octa-*O*-benzylated procyanidin B3, by which the condensation reaction between two benzyl substituted (+)-catechin units, 5,7,3’,4’-tetrabenzylcatechin (as a nucleophile) and (2*R*,3*S*,4*S*)-5,7,3’,4’-tetrabenzyloxy-3-acetoxy-4-methoxyflavan (as an electrophile) was carried out in the presence of TiCl_4_ to synthesize octabenzylated procyanidin B3 efficiently (83%) and stereoselectively (4α:4β = 23:1) [50]. Octa-*O*-benzylated procyanidin B3 was also successfully synthesized with high levels of stereoselectivity (4α:4β = 66:1) and in excellent isolation yields of gram-scale, through the condensation reaction between the nucleophilic 5,7,3’,4’-tetrabenzylcatechin and the electrophilic (2*R*,3*S*,4*S*)-3-acetoxy-5,7,3’,4’-tetrabenzyloxy-4-(2’’-ethoxyethoxy)flavan in the presence of TMSOTf at -78°C. This resulted in an octabenzylated procyanidin B3 that could be converted to its natural form efficiently by using hydrogenolysis chemicals, as shown in Scheme 5 [51]. 

**Scheme 6:**
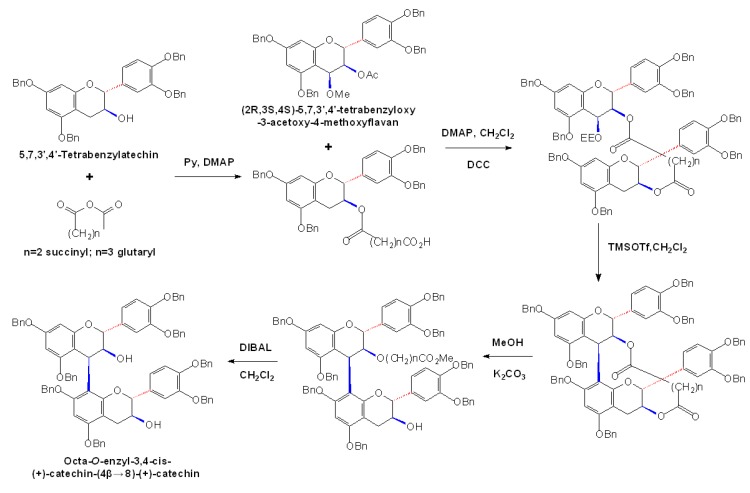
TMSOTf-catalyzed intramolecular condensation [52].

Interestingly, a new TMSOTf-catalyzed intramolecular condensation method was subsequently developed, by which a potential electrophile and a nucleophile were connected with diester linkers at high reaction temperature, and the resulting condensed product was then transformed into the natural (+)-catechin-(4β→8)-(+)-catechin dimer, as shown in Scheme 6. Although in need of more complex reaction processes, this method used just equimolar amount of nucleophile and electrophile in good yield and its main product with 3,4-cis configuration was even rare in nature [52]. Also with the aid of TMSOTf, (2*R*,3*R*,4*S*)-5,7,3’4’-tetra-*O*-benzyl-4-(2’’-ethoxyethyloxy)flavan derived from (-)-epicatechin was used as an electrophile to react with the dimeric nucleophiles, producing four benzylated natural procyanidin trimers which contained (-)-epicatechin as the upper unit, including procyanidin C1, C4 and their analogues, (-)-epicatechin-(4β→8)-(-)-epicatechin-(4β→8)-(+)-catechin and (-)-epicatechin-(4β→8)-(+)-catechin-(4α→8)-(-)-epicatechin [53].

**Scheme 7:**
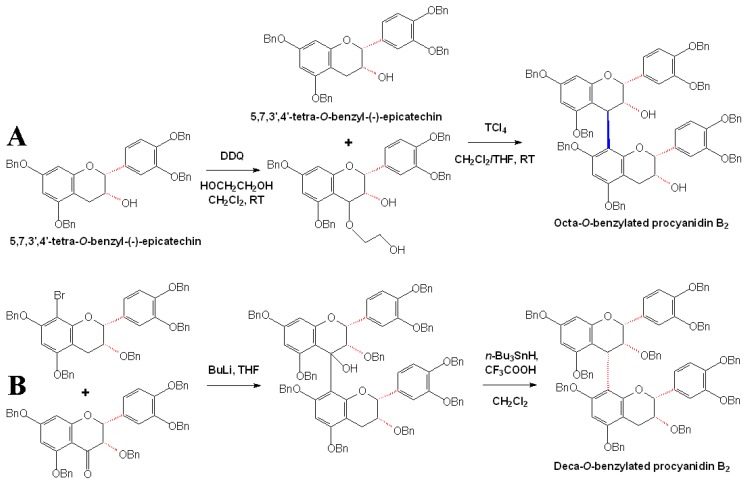
Stereoselective assembly of benzylated procyanidin B2 (A) and its stereoisomer (B) [54,55].

Almost meanwhile, a partially optimized procedure for stereoselective synthesis of procyanidin B2 was also developed. Firstly, 5,7,3’,4’-tetra-*O*-benzyl-(-)-epicatechin derivated from (+)-catechin was obtained by benzylation of the phenolic oxygens and the transformation of the configuration at the C3 position as a nucleophilic building block, which was further transformed to an electrophilic building block by the oxidation with DDQ. The TiCl_4_-mediated condensation of the two substrates permitted the stereoselective assembly of the octabenzylated (-)-epicatechin oligomer, procyanidin B2 (Scheme 7A) [54]. By using the developed methods, a highly stereoselective synthesis of a stereoisomer of procyanidin B2, epicatechin-(4α→8)-epicatechin, was obtained and this stereoisomer was rare in nature and had not been accessible through stereoselective synthesis until that time (Scheme 7B) [55]. 

**Scheme 8:**
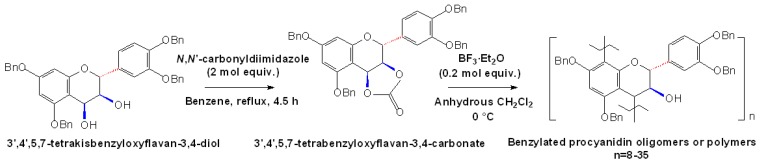
Stereoselective synthesis of PAs with high molecular mass [47].

The synthesis of bis(5,7,3’,4’-tetra-*O*-benzyl)epicatechin 4β→8-dimer from 5,7,3’,4’-tetra-*O*-benzylepicatechin and 5,7,3’,4’-tetra-*O*-benzyl-4-(2-hydroxyethoxy)epicatechin was further improved by replacing the Lewis acid TiCl4 employed previously with the clay mineral Bentonite K-10 [56]. With the same conditions, the benzyl-protected trimeric, tetrameric, and pentameric procyanidin oligomers (DP≤5) containing all 4β→8 IFL were obtained regioselectively from their lower homologues. Besides the synthesis of procyanidin oligomers, the condensed tannins with high molecular mass were obtained by the cationic polymerization of 3’,4’,5,7-tetrabenzyloxyflavan 3,4-carbonate and subsequent debenzylation, as shown in Scheme 8. Using this strategy, a series of PAs with high molecular mass (the highest average molecular mass: 10,200) and high degree of polymerization (the highest degree of polymerization was 35) were synthesized, which could be used as model compounds for natural condensed tannins [47].

**Scheme 9:**
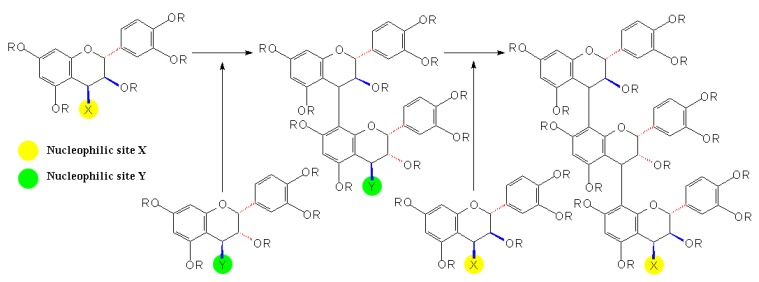
Orthogonal strategy for stereoselective synthesis of PA oligomer [48].

More recently, enlightened in particular by a potential orthogonal strategy for oligosaccharide synthesis, Ohmori *et al*. developed a new orthogonal synthetic strategy for PAs, which enabled the controlled formation of oligomeric (+)-catechins [48]. The bromo-capping of the C8 position of the flavan skeleton, the selection of the substitute of the C4 position and the hard or soft activation of Lewis acid enabled the equimolar coupling of electrophilic and nucleophilic (+)-catechin derivatives, avoiding the self-condensation of the substrates and enabling practical and reiterative chain extension of (+)-catechin units to synthesize complex (+)-catechin oligomers [48]. Therefore, orthogonal strategies for stereoselective synthesis of PA oligomers with diverse flavan-3-ols can be easily developed (Scheme 9).

### 2.4 Synthesis of A-type Procyanidins

Although the chemical synthesis of B-type procyanidins has been investigated widely and deeply, there are few reports about the synthesis of A-type procyanidins. Some researchers suggested that A-type dimeric or oligomeric PAs could be formed *in vitro* by direct condensation between anthocyanin and (+)-catechin or (-)-epicatechin, which was supported by the formation of a series of colorless bicyclic anthocyanin-flavan-3-ol dimer containing both C4-C8 and C2-O-C7 ether type IFL [57,58]. However, these oligomers are different from their natural analogues, because they usually contains additional methl groups at C3’ and/or C5’ positions and glucosyl groups at C3 and/or C5 positions. Until now, it almost remains no report about the synthesis of A-type procyanidins by direct condensation of (+)-catechin (and/or (-)-epicatechin) or their derivatives.

**Scheme 10:**
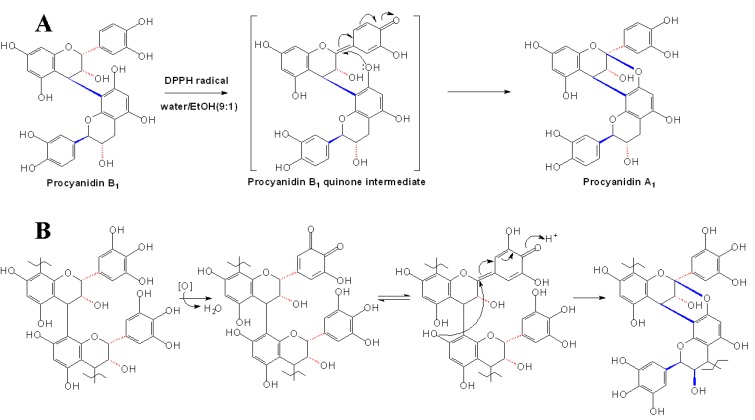
DPPH (A) and PPO (B) mediated transformation of B-type procyanidins to A-type procyanidins [60,61].

On the other hand, A-type procyanidins can be formed from the B-type procyanidins *in vitro*. Burger *et al*. first found that an initial oxidative removal of hydride ion at C2 position could result in the oxidative transformation of B-type procyanidins into A-type procyanidins [59]. Subsequently, procyanidins B1 and B2 were respectively converted into procyanidins A1 and A2 by radical oxidation using 1,1-diphenyl-2-picrylhydrazyl (DPPH) radicals under neutral conditions, which was involved in the abstraction of the hydrogen atom at the C2 position (Scheme 10A) [60]. Further more, the studies on the oxidation of (-)-epicatechin in banana extracts indicated that the A-type IFL of the PA was probably formed by polyphenol oxidase (PPO) mediated oxidation of the B-ring of the upper unit and subsequent addition of the C7 hydroxyl group of the lower unit to the C2 position of the resulting quinonoidal structure (Scheme 10B) [61]. However, in the larger oligomeric A-type PAs which contain both A-type IFL and B-type IFL, we still do not know the exact assembly or formation sequence of the two kinds of IFL.

## 3. Reactions of Proanthocyanidins in Wine

### 3.1 Proanthocyanidins in wine

PAs are one group of important polyphenolic compounds in wine, which are extracted from wine grapes during winemaking operations (crushing, maceration, and fermentation), and play a significant role in the organoleptic evaluation, such as eliciting persistent bitterness and astringency [6,62]. The total PA content of red wines, averaging around 175 mg/L, is approximately 20-fold higher than that of white wines because of the different grape material use and different enology technologies [63]. Another group of important polyphenolic compounds in wine involves anthocyanins, which mainly provide the red color in red wine, but also have a slight contribution to the astringency [64].

Astringency and bitterness are two of the main sensory attributes of wine [65]. Generally for human beings, bitterness is perceived by taste receptors on the tongue and astringency is a tactile sensation perceived as dryness, puckering and roughness throughout the oral cavity, all of which could be offered by PAs in wine due to their polyhydric structure and the protein-binding ability [66,67,68]. Furthermore, the nature of PAs, including the molecular sizes, the kinds of IFL and especially the monomeric composition, has a great influence on the sensation of bitterness and astringency. For example, as the chain length of PAs increases (up to decamer level or higher until the polymers become insoluble in solutions), the astringency increases, whereas the bitterness decreases. The oligomers with the C4→C6 IFL are more bitter than the oligomers linked by the C4→C8 IFL [69]. On the other hand, (-)-epicatechin is higher in astringency and bitterness than (+)-catechin, and the oligomers with more composition of (-)-epicatechins are higher in astringency than the ones with more composition of (+)-catechin. Also, the greater percentage of galloylation in PAs will provoke a greater sensation of astringency [70].

The nature of PAs in wine strongly depends on their origin, the grape berries. In grape berries, PAs are mainly presented in B-type oligomers or polymers and are localized in the solid parts, and several recent reports reveal the evidence for the existence of A-type PAs and their galloylated derivatives [71]. Seed PAs consist of (+)-catechin, (-)-epicatechin, and (-)-epicatechin-3-*O*-gallate subunits. Skin PAs also contain (-)-epigallocatechin and small amounts of (-)-epicatechin-3-*O*-gallate. Stem PAs are made up of all of the four main monomers: (+)-catechin, (–)-epicatechin, (–)-epigallocatechin and (-)-epicatechin-3-*O*-gallate subunits. Thus, seed PAs only contain procyanidins or their derivatives, while skin and stem PAs contain both procyanidins and prodelphinidins. Further more, the PA amount, composition, and mean degree of polymerization (mDP) differ among berry skins, seeds and stems. For instance, skin PAs have a higher mDP and a lower proportion of galloylated subunits than those from seeds, and the mDP of stem PAs is similar to those of seed PAs, but sometimes seems to be even higher than that of skin PAs. However, anthocyanins are only present in grape berry skins [72].

During the storage and aging of red wines, the composition of PAs and anthocyanins change continuously, leading to the loss of astringency and the change in color from red to tawny. Such changes are closely related to the dissociation and polymerization of PAs, the formation of higher molecular weight PA-anthocyanins, the condensation of anthocyanins and flavanols and even other more complex chemical reactions [73]. It is worth of pointing out that polymeric anthocyanins are suggested to be responsible for the stable color of red wines, which is more stable to pH increases than monomeric anthocyanins, and are also less sensitive to oxidation and to bleaching by sulphur dioxide than monomeric anthocyanins [14,74].

### 3.2 Direct reactions among flavanols, anthocyanins and proanthocyanidins

It was ever thought that oligomeric PAs would undergo further condensation with their monomers or anthocyanins during storage and aging of red wine, to form higher molecular weight polymers, which led to their eventual precipitation and the loss of the bitterness and astringency [69,75]. However, this suggestion has been altered due to the finding that higher molecular weight PAs (DP>20) were still soluble in wine and were possible particularly astringent [76,77]. More and more studies have indicated that reactions of PAs in wine will produce both larger polymers and smaller species, which are involved in both the acid-catalyzed C-C bond-breaking and bond-making process. To some extent, the reduction rather than the increase of the average molecular weight of PAs may be more responsible for wine deastringency [78]. 

**Scheme 11:**
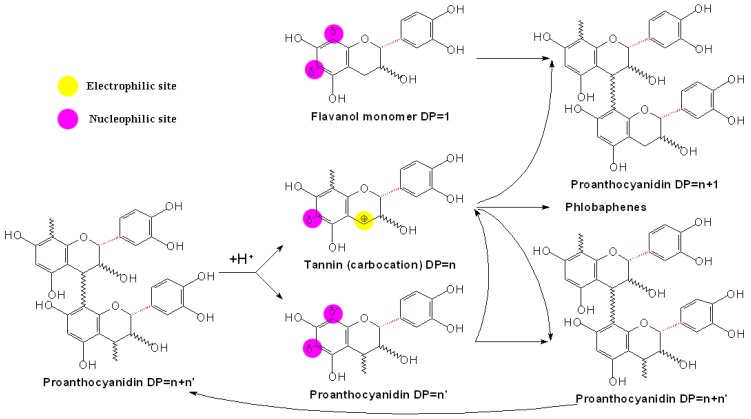
Direct reactions between flavanols and PAs in wine [79].

To support this, research was conducted in wine-like model solutions involving reactions of seed and skin PAs in the presence or absence of (-)-epicatechin, which showed interesting results. In the solutions containing no free (-)-epicatechin, trace amounts of monomers were released and important losses of PAs were measured, but their average composition and mDP were hardly modified. However, in the solutions containing free (-)-epicatechin, the mDP value decreased dramatically and oligomeric PAs accumulated while losses of total units were noticeably reduced. The results above demonstrate that under mild acidic conditions as encountered in wine, PAs undergo spontaneous cleavage of their IFL to release unknown carbocations, which led to the loss of the total composition of PAs, and on the other hand, the released carbocations could be attacked by the nucleophilic units, such as (+)-catechins, (-)-epicatechins or their small oligomers, to form new and shorter PAs, which resulted in the reduction of the average chain length of PAs and the ultimate accumulation of oligomeric PAs, as shown in Scheme 11. Further more, the rate of the C-C bond-breaking process was primarily determined by pH, and the kinetics of subsequent reactions was thought to depend on the ratio of polymers to monomeric species [79].

Besides flavan-3-ols [mainly (+)-catechins or (-)-epicatechins], anthocyanins can also act as nucleophiles and compete with flavanols to condense with the carbocations to form further polymeric pigments [80]. For instance, PA-anthocyanidin^+^ (T-A^+^) pigments with lower molecular weight occurred in the direct reaction of PAs and anthocyanins in wine. In this reaction, the electrophilic units are the carbocations resulting from cleavage of PAs, which reacts with the C8 or C6 position of additional anthocyanins in their nucleophilic hydrated forms. The resulting flavanol hemiketal adducts (T-AOH) can be converted into the corresponding flavylium form (T-A^+^) under the wine pH. On the other hand, anthocyanidin^+^-PA (A^+^-T) adducts are also found in the fractions containing polymeric PAs. In this kind of reactions, the electrophilic C4 positions of the flavylium from anthocyanins (A^+^) are attacked by the nucleophilic C8 or C6 positions of the flavanol units of additional PAs to yield an A^+^-T adducts [58]. Thus, both types of anthocyanidins and PAs can act either as a nucleophile or as an electrophilic species, and the two mechanisms differ in the position of anthocyanins in the resulting products.

**Figure 1:**
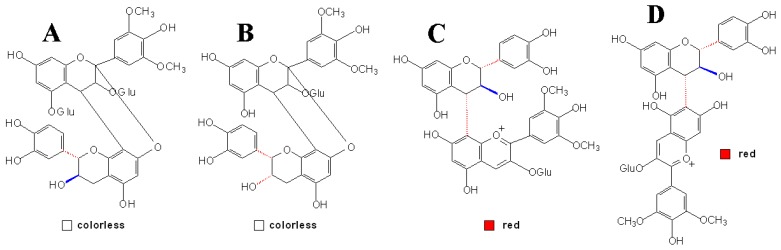
Structures of the condensed products of anthocyanin and flavan-3-ols [57,58,81].

Direct reactions can also occur between anthocyanins and flavanols, which have two mechanisms leading to the formation of anthocyanin-flavanol (A^+^-F) and to the formation of flavanol-anthocyanin (F-A^+^) adducts, respectively, and are similar to the direct reactions between PAs and anthocyanins [81]. Some researches indicated that these flavene adducts have been oxidized into the corresponding flavylium, which in turn proceeded to the yellow xanthylium ion, explaining the formation of yellowish pigments appeared in solutions containing grape anthocyanins and flavanols [82]. However, other research indicated that xanthylium derivatives were from the glyoxylate-mediated condensation of flavanols with both no involvement of anthocyanins and no xanthylium pigments but flavylium derivatives would be formed after the direct condensation of anthocyanins and flavanols [83]. To date, this argument still continues.

In the early researches of this field, Jurd found that in the oxidative condensation of flavylium salts and (+)-catechins, a crystalline 4-flavanylflavylium salt was first formed and then was then oxidized by a second molecule of the flavylium salt, which is reduced to a monomeric flav-2-ene [84]. The reaction of malvidin 3,5-diglucoside and (+)-catechin in aqueous 10 mM HCl could form an exclusive colorless, C44, bicyclic condensation product, which was rare in nature at that time and even now, as shown in Figure 1A [57]. More recently, the products from direct reaction of malvidin 3-glucoside and (-)-epicatechin in ethanol were postulated to be colorless, bicyclic dimers linked with both carbon-carbon and ether bonds as observed in the case of A-type PAs, and the major dimmer analyzed by NMR experiments was identified as malvidin 3-glucoside-(C2→O→C7, C4→C8)-(-)-epicatechin (Figure 1B). And also, similar results were obtained in the condensation of malvidin 3-glucoside with (+)-catechin instead of (-)-epicatechin in ethanol [58]. 

However, some other researches showed that the direct reaction of malvidin 3-glucoside and flavanol only formed colorless dimers, linked by only carbon-carbon IFL which seemed not to evolve towards the formation of pigments of tawny-yellowish tonality [85]. In the interaction also between (+)-catechin and malvidin-3-glucoside, the anthocyanin in flavylium form would bind through its C4 position to the C8 position of a followed catechin to give a colorless condensed flavene, which would later become rearranged to a xanthylium-like derivative through an oxidative mechanism [82]. Nevertheless, in the investigation of Salas *et al*., red (+)-catechin-(4α→8)-malvidin 3-glucoside and (+)-catechin-(4α→6)-malvidin 3-glucoside were obtained from the reaction of taxifolin and malvidin 3-glucoside following a protocol adapted from PA dimer synthesis (Figure 1C, D) [81]. These investigation extended our eyeshot of the direct reaction between anthocyanins and flavanols, and also gave us an opportunity to further the color properties of the flavanol-anthocyanin pigments, for example, ruling out the assumption that flavanol-anthocyanin adducts are more resistant to hydration and sulfite bleaching than anthocyanins monomers.

Another important direct reaction is concerning the autoxidation of flavanols, especially (+)-catechin and/or (-)-epicatechin, offering the unnatural oligomers of these flavan-3-ols, which have the same molecular weight as their natural isomers, procyanidins, but have completely different structures [86]. Normally, the oxidation dimers of flavan-3-ols linked by alternative C6’→C8 or C6’→C6 IFL are classified as B-type dehydrodicatechins, which are colorless. And correspondingly, the oxidation dimers which contain additional C-O-C ether-type IFL are classified as A-type dehydrodicatechins, which are usually yellow [87]. Although the oxidation of flavan-3-ols can occur very fast under acidic conditions, especially when metal catalysts are present, such as the condition of wine, such oxidation oligomers of flavan-3-ols have not been identified or isolated from wines until now [86,88]. This may be due to the poor production of the autoxidation and some other reactions in wine which probably compete with this autoxidation reaction or even restrain it.

### 3.3 Indirect reactions among flavanols, anthocyanins and proanthocyanidins

In the research on the interactions among anthocyanins, phenolic compounds, and acetaldehyde in red wine, researchers found that the addition of acetaldehyde to the mixtures of anthocyanins and phenolics caused rapid and spectacular color augmentation with shifts toward violet, with extent of the shift varying with the type of component [89]. They supposed that this color augmentation was due to the formation of highly colored new compounds which consisted of anthocyanins and phenolics linked by CH_3_CH bridges [90]. Since then, a great deal of efforts have been put into the investigation of the acetaldehyde-mediated polymerization of flavanols, anthocyanidins, and PAs, and more and more studies have been published to support this assumption.

**Figure 2:**
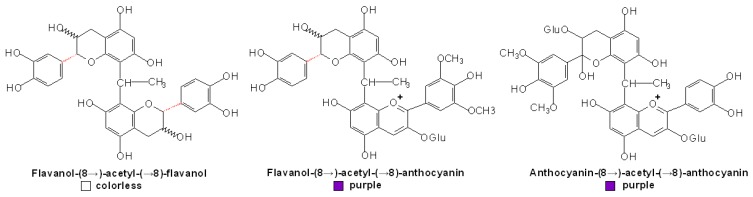
Ethyl-bridged dimers of flavan-3-ols and anthocyanins [93,94,95].

Generally, besides the direct reaction of flavanols, anthocyanidins, and PAs, another main mechanism of their polymerization in wine is mediated by acetaldehyde. Acetaldehyde is the most abundant aldehyde present in wine and would arise from different sources such as a fermentation intermediary product, from oxidation of ethanol, or from the addition of wine spirit to the must in order to stop the fermentation [91,92]. In acidic media such as wine, after the protonation of acetaldehyde, the flavanols can undergo electrophilic substitution by acetaldehyde on the nucleophilic C6 or C8 positions of their A-ring, giving rise to the formation of condensation products which are colorless when derived from only flavanols, or are reddish or violet when anthocyanins participate in (λ_max_ 528~540 nm, purple), as shown in Figure 2 [93,94,95]. These substances are transient and they later evolve to form substances with a greater degree of condensation, which finally precipitate [96].

Until now, various products of the acetaldehyde-mediated polymerization of them in wine have been reported, including homogeneous ethyl-bridged oligomers of (+)-catechins or (-)-epicatechin, heterogeneous ethyl-bridged oligomers of (+)-catechins with (-)-epicatechins, heterogeneous ethyl-bridged oligomers of (+)-catechins or (-)-epicatechin with malvidin 3-glucoside, or even heterogeneous ethyl-bridged oligomers consisting of oligomeric procyanidins and malvidin 3-glucoside linked with an ethyl bridge. However, the ethyl-bridged units can link through their C6 or C8 positions on the A-ring, yielding four different linking patterns C6-C6, C8-C8, and C6-C8 (R and S) linkages (the presence of an asymmetric carbon for the C6-C8 isomers) [97]. Thus, the acetaldehyde-mediated polymerization with simple precursors usually produces oligomers with complex structures. For example, Saucier *et al*. synthesized homogeneous (+)-catechin-(6→)-acetyl-(→8)-(+)-catechin dimers and (+)-catechin-(8→)-acetyl-(→8)-(+)-catechin-(6→)-acetyl-(→8)-(+)-catechin-(6→)-acetyl-(→8)-(+)-catechin tetramers, Rivas-Gonzalo *et al*. synthesized heterogeneous (+)-catechin-(8→)-acetyl-(→8)-malvidin 3-glucoside, and Es-Safi *et al*. obtained a series of homogeneous or heterogeneous ethyl-bridged oligomers through the condensation of (+)-catechin and (-)-epicatechin with acetaldehyde [96,97]. It is important that malvidin 3-glucosides only show at the terminal points of the liner oligomers linked by acetyl, thus no compound containing more than two malvidin 3-glucoside units is present [98]. On the other hand, the polymerization of flavanols, dimeric, galloylated dimeric and trimeric procyanidins with malvidin-3-glucoside in the presence of acetaldehyde showed different reaction speeds, generally, decreasing as the order followed, procyanidin C1, procyanidin B1, procyanidin B2, procyanidin B2-3’-*O*-galloyl, procyanidin B3, (-)-epicatechin and (+)-catechin. [99]

Besides acetaldehyde, other aldehydes can also mediate the condensation of flavanols, anthocyanidins, and PAs in wine, such as isovaleradehyde, benzaldehyde, propionaldehyde, isobutyraldehyde, formaldehyde, 2-methybutyraldehyde, etc. [100]. For example, a propionaldehyde mediated condensation reaction between malvidin 3-glucoside and (+)-catechin was found to lead to the formation of malvidin 3-glucoside-(8→)-propyl-(→8)-(+)-catechin [101]. In the presence of furfural or its derivative 5-(hydroxymethyl) furfural, the reactions between (+)-catechin and anthocyanins were also detected to produce various furfuryl or 5-hydroxymethylfurfuryl group linked oligomers [102]. In addition, xanthene and xanthylium salts derivatives were obtained from (+)-catechin-furfuryl/-hydroxymethylfurfuryl-(+)-catechin and (+)-catechin-furfuryl/-hydroxymethyl-furfuryl-malvidin 3-glucoside dimers, through dehydration and oxidation. Therefore, these reactions may contribute to the decrease of astringency and the change of color observed during the aging of grape-derived foods, especially in wines.

**Scheme 12:**
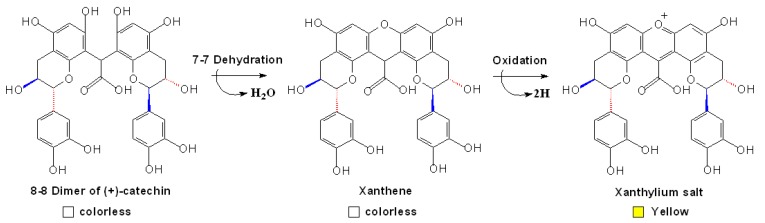
Formation of xanthylium salt derivated from glyoxylic acid-mediated dimeric (+)-catechin [83,105].

Glyoxylic acid-mediated polymerization of flavanols is another indirect condensation mechanism which was found from wine in recent years [103]. Similar to the acetaldehyde-mediated polymerization, glyoxylic acid from iron-catalyzed oxidation of tartaric acid attacks the nucleophilic C8 or C6 positions of two flavanols to produce a carboxy methane-bridged dimer that exhibits maxima absorbance at 280 nm, and this dimer can be dehydrated to a xanthene derivative and then be oxidated into a xanthylium salt (λ_max_ 440~460 nm, yellow), as shown in Scheme 12 [104,105]. Considering the relative amounts of the tartrate acid and ferrous ions in wine, this reaction may be an important route during wine aging, which probably competes with the acetaldehyde-mediated polymerization and contribute considerable yellow color to the aging wine [106].

### 3.4 Other Anthocyanins Derived Condensed Pigment

**Figure 3:**
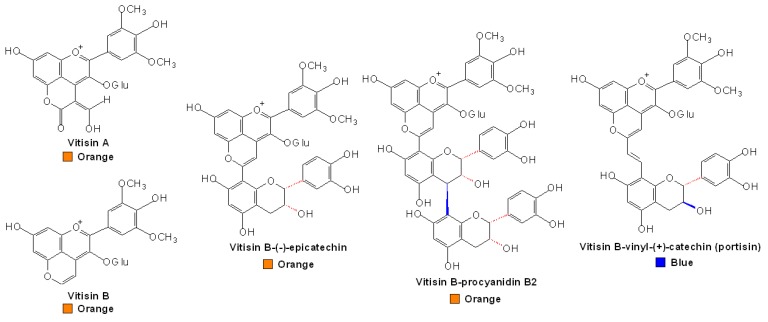
Structures of pyranoanthocyanins [108,110,111].

A new group of red anthocyanin-derived pigments, pyranoanthocyanins, have been identified in wine, and these pigments are proved to be more stable to be resistant to bleaching by sulfur dioxide and provide a great contribution to the wine color (λ_max_ 490~510 nm, orange), especially in the storage and aging of wine [107]. Their formation results from cyclization between C4 position and the hydroxyl group at C5 position of the original flavylium moiety of the anthocyanin with the double bond of the adduct, followed by dehydration and rearomatisation steps to form the final configuration of the new pigments, some of which were named vitisin by Bakker and Timberlake [108]. In their research, four vitisin-like pigments were found in trace amounts in red wine during maturation, which were further isolated and identified as the vitisin A, acetylvitisin A, vitisin B and acetylvitisin B (Figure 3).

To date, more and more substances have been found to be involved in the formation of vitisin-like pigments as adducts, such as pyruvic acid, acetaldehyde, 4-vinylphenol and ethylflavanol [109]. On the other hand, these formed pyranoanthocyanins can further condense with monomeric flavan-3-ols or oligomeric PAs to produce bigger and more stable pigments. In the model solutions containing malvidin 3-glucoside, flavan-3-ols and procyanidin B2 in the presence of acetaldehyde, a group of new orange-red pigments were identified, in addition to some usual compounds such as an ethyl bridge-linked malvidin 3-glucoside with procyanidin B2. This new pigment was speculated to be formed by reaction of the malvidin 3-glucoside cation with a procyanidin B2 moiety (Figure 3) [110]. Interestingly, a new class of vinylpyranoanthocyanin pigments (λ_max_ 575 nm, blue), named portisins, were also isolated and structurally characterized more recently, which are proposed to result from the condensation between anthocyanin-pyruvic acid adducts and flavan-3-ol adducts by a vinyl bridge, as shown in Figure 3 [111,112].

However, during the storage and aging of wine, anthocyanins undergo various reactions which can generate colorless or colored as well as polymeric and small various pigments [14,73]. And also, some polymeric pigments undergo sulfite bleaching while some low molecular weight pigments do not, all of which have a great influence on the color and flavor of the wine [113].

## 4. Conclusions and Perspectives

During the past several decades, chemical synthesis of PAs has developed greatly. Using flavan-3,4-diols or their derivatives as electrophile and flavan-3-ols as nucleophile, the technologies of biomimetic or chemical synthesis show us an operational platform to study the potential mechanism of PA polymerization and offer us a route to obtain various PAs in the artificial system. With a series of residue protection and activation methods, the technologies of stereoselective synthesis offer us the possibility to synthesis the specific PA with high efficiency and stereospecificity, which can be further applied as chemical standards or bioactive supplements. On the other hand, research on the direct or indirect PA-involved reactions offer us a systematic and comprehensive understanding about the alteration of polyphenolic chemicals during wine storage or aging, especially the changes of PAs, which help us to improve the technologies of enology to produce wines with high quality, and also show us an opportunity to investigate the chemical synthesis of PAs in another visual angle. However, in these research fields, there are still some future objects as follows:

1) Synthesis of derivatives of flavan-3,4-diol with balanced electrophilicity and stability which can be obtained commercially and can be used in the chemical synthesis of PAs directly.

2) Synthesis of PAs with different hydroxylation patterns, which can be used as heterocyclic chemicals for their bioactive benefits.

3) Stereoselective synthesis of PAs with high production, high efficiency and high stereospecificity, as well as PAs with high chain length and high molecular weight.

4) Discovery of new Lewis acids used in stereoselective synthesis of PAs, which allows high reaction temperature and short reaction time.

5) Development of the new technologies of enology, which leads to the maintenance of appropriate PAs with suitable chain length and flavor in wine even after long time of aging.

6) Systematic research of the anthocyanin involved reactions in wine aging, and development of the new technologies of enology to enhance the color and its maintenance.

7) Search the condensed pigments with particularly new structures which are in trace amounts but have considerable contribution to the wine color.
